# Inflammatory Myalgia, A Rare Presentation of Hodgkin's Lymphoma: About a Case

**DOI:** 10.7759/cureus.47379

**Published:** 2023-10-20

**Authors:** Ilyass Chergaoui, Anas Kherrab, Mirieme Ghazi, Abderrahim Raissi, Mohamed Amine Azami, Redouane Niamane

**Affiliations:** 1 Department of Rheumatology, Military Hospital Avicenne, Marrakech, MAR; 2 Department of Hematology, Military Hospital Avicenne, Marrakech, MAR; 3 Department of Pathology, Caddi Ayyad University of Marrakech/Ibn Sina Military Hospital, Marrakech, MAR

**Keywords:** magnetic resonance imaging, hodgkin lymphoma, muscle aches, 18f-fluorodeoxyglucose positron emission tomography (18f-fdg pet), myalgia

## Abstract

Hodgkin's lymphoma (HL), also known as Hodgkin lymphoma or Hodgkin disease, is a type of malignancy that originates in B lymphocytes, which are a type of white blood cells involved in the immune system. It is characterized by the presence of abnormal Reed-Sternberg cells within the lymph nodes or other lymphoid tissues. Bone involvement of HL is exceptional, which can be localized or part of a disseminated disease. The case of our patient is a Hodgkin's lymphoma initially presenting with a complaint of myalgia. Magnetic resonance imaging and 8F-fluorodeoxyglucose positron emission tomography (FDG PET) played a crucial role in the diagnosis of this rare case.

## Introduction

Hodgkin's lymphoma (HL) is a malignant B-cell lymphoid hemopathy. The disease shows a bimodal distribution, with the majority of cases occurring between 15 and 30 years of age, followed by another peak in adults aged 55 years or older. The phenotype of HL emerges at the crossroads of inflammation and malignancy, making it unique and distinct when compared to other malignancies. The disease primarily affects cervical, supraclavicular, and mediastinal lymph nodes. Bone marrow and hepatic involvement is less frequent.

Muscle involvement during this pathology is rare and is observed in less than 1.5% of patients [1]. Similarly, bone involvement of LH is exceptional, which can be primitive or part of a disseminated disease [2]. We report the case of a patient with atypical Hodgkin's lymphoma revealed by myalgia.

## Case presentation

A 21-year-old woman, with no significant medical history, presented with myalgia evolving for two months and involving the two thighs, with inflammatory pain exacerbated by physical exertion.

On clinical examination, the patient had a waddling gait due to the thigh pain, with a motor function deficiency involving the thighs, arms, and forearms. There was no muscular atrophy. The lymphoreticular and neurological examinations were normal.

The biological assessment reveals an inflammatory biological syndrome with a C-reactive protein (CRP) level of 161 mg/l, erythrocyte sedimentation rate (ESR) of 110 mm during the first hour, anemia (hemoglobin of 10.9 g/dL), and leukocytosis (12.103/μL) with neutrophil predominance. The quantification of muscle enzymes was normal (lactate dehydrogenase (LDH) at 197 U/I and creatine phosphokinase (CPK) at 20 U/I). This assay was repeated three times without abnormality. The cortisol level was normal (99 µg/L). The levels of calcemia and angiotensin-converting enzyme (ACE) were normal. The serum electrolyte test and liver tests were normal (Table 1). An assessment for the detection of infections was performed, including procalcitonin, the detection of Mycobacterium tuberculosis, urine culture, hepatitis B/C, HIV, syphilis, influenza virus, Cytomegalovirus (CMV), Epstein-Barr virus (EBV), Coxsackie, trichinosis, toxoplasmosis, cysticercosis, and severe acute respiratory syndrome coronavirus 2 (SARS‑CoV‑2); all were negative.

**Table 1:** The patient's laboratory findings CRP: C-reactive protein; ESR: erythrocyte sedimentation rate; LDH: lactate dehydrogenase; CPK: creatine phosphokinase; ACE: angiotensin-converting enzyme

Laboratory findings	Patient values	Normal values
CRP	161 mg	< 4 mg/l
ESR	110 mm	< 20 mm
Hemoglobin	10.9 g/dl	13 g/dl
Leucocyte count	12,103 / μL	4,000 – 10,000 /μL
LDH	197 U/I	135 – 214 U/I
CPK	20 U/I	< 200 U/I
Cortisol	99 μg/L	62-194 μg/L
Calcemia	2.20 mmol/l	2.12-2.55 mmol/l
ACE	24 U/L	13.3–63.90 U/L

The immunological assessment was negative (anti-cyclic citrullinated peptide (CCP) antibodies, latex Waaler-Rose, antinuclear antibodies, anti-native DNA antibodies, anti-soluble antigen antibodies and anti-synthetase antibodies, anti-Jo1 antibodies, anti-PL7 antibodies, anti-PL12, anti-EJ antibody, anti-OJ antibody, anti-Mi2 antibody, anti-Ku antibody, anti-PM100 antibody, anti-PM75 antibody, anti-SRP antibody, and antiRo-52 antibody).

**Figure 1:**
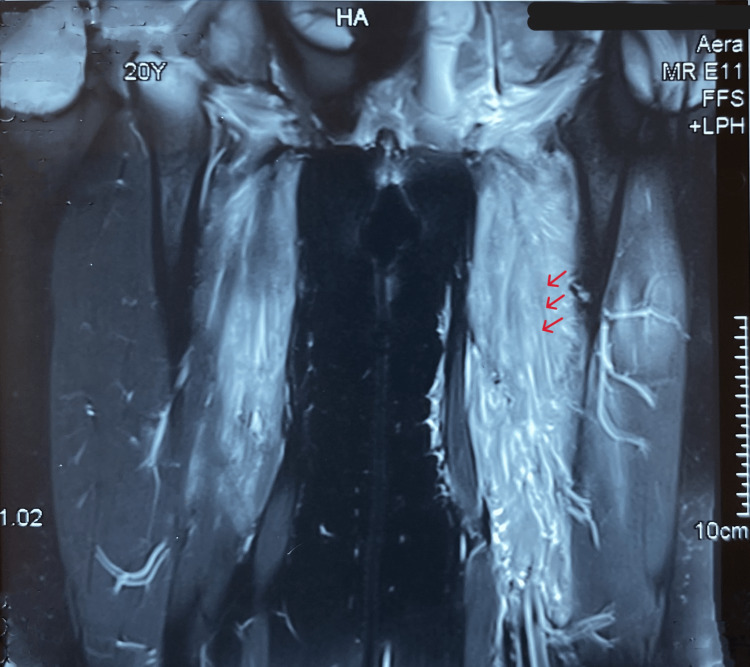
MRI of the thighs shows an inflammatory myositis of the thighs, with edematous infiltration of the muscles of the two thighs involving the short and long adductor muscles and the left quadriceps extending to the intermuscular fascias in hyposignal T1 and hypersignal T2. (red arrows)

Thoracic CT demonstrated several mediastinal adenopathy, measuring 12.6 mm for the largest. MRI of the thighs showed an aspect of inflammatory myositis of the thighs (Figure 1). The electromyography of the lower limbs indicates signs of myogenic involvement. The muscle biopsy shows inflammatory myositis and fibrosis with a discrete diffuse mononuclear cell infiltration. The minor salivary gland biopsy shows no signs of Sjögren’s syndrome, sarcoidosis, or amyloidosis.

The patient was treated with oral glucocorticoids (prednisone 20 mg/day), with a partial response that lasted 20 days. A month later, the patient continued to experience inflammatory myalgia in both thighs, with the persistence of the biological inflammatory syndrome.

On the other hand, the serial follow-up thoracic CTs showed a regression of the mediastinal adenopathies. The patient received IV methylprednisolone at a dose of 240 mg per day for three days, and the prednisone was increased to 60 mg/day with no clinical improvement.

**Figure 2:**
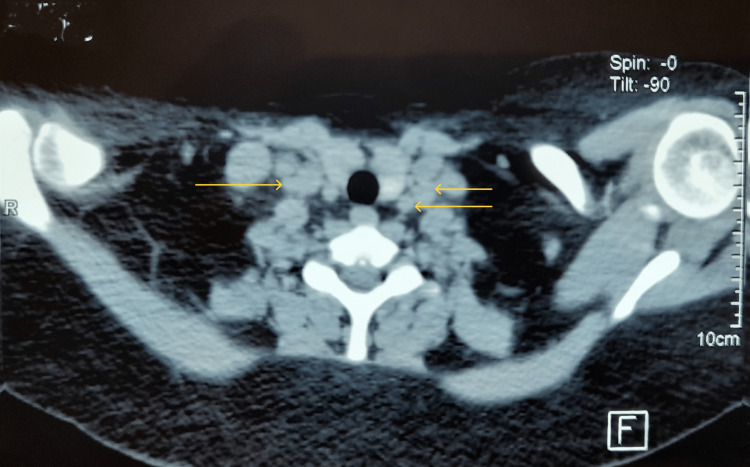
Thoracic CT showing the mediastinal adenopathies (arrows)

The patient showed the presence of jugular carotid adenopathy on the right side (measuring 13 x 15 mm / 21 x 10 mm), right spinal adenopathy (12 x 17 mm and 19 x 15 mm), and laterotracheal mediastinal adenopathy on the left side (19.6 x 11 mm) and in the right pre-tracheal space (15 x 9.6 mm and 14 x 12 mm) (Figure 2).

**Figure 3:**
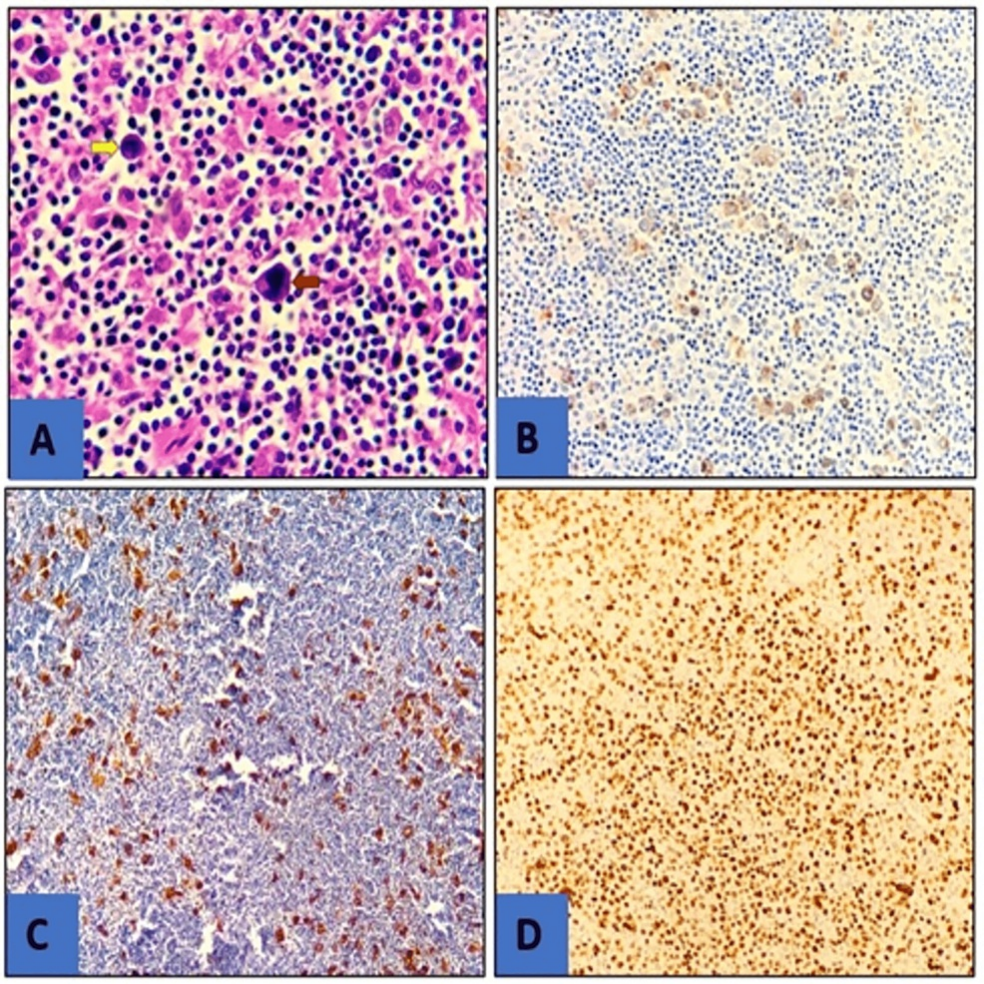
Photomicrograph image showing histology with the hematoxylin and eosin (HE) stain and immunohistochemical features of classical Hodgkin lymphoma (A) (HE x 200) showing the presence of Reed Sternberg's binucleated tumor cells (red arrow) and Hodgkin's mononuclear tumor cells (yellow arrow) within a reactive inflammatory background. Immunohistochemistry findings (X 200); (B) positivity for CD30; (B) positivity for CD15; (C) positivity for Pax5

**Figure 4:**
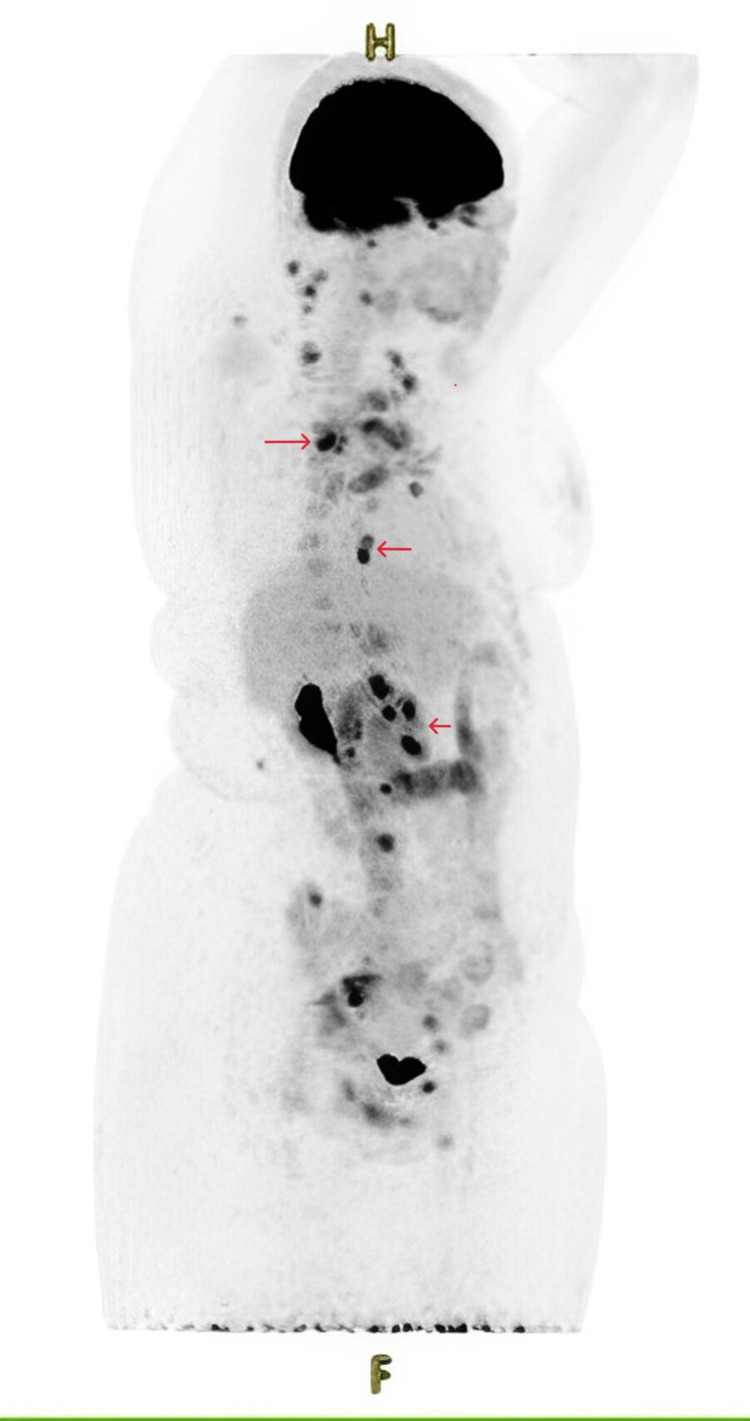
FDG PET shows stage IV of the Ann Arbor classification of Hodgkin lymphoma with diffuse involvement of the extralymphatic organs, with associated lymph node involvement (arrows) FDG PET: 18F fluorodeoxyglucose positron emission tomography

The mediastinal adenopathy biopsy revealed nodular sclerosis classical Hodgkin lymphoma (NSCHL) (Figure 3). FDG PET (18F fluorodeoxyglucose positron emission tomography) scan confirmed stage IV Hodgkin's lymphoma with cervical, mediastinal, abdominal, and right femoral diaphyseal location (Figure 4).

The patient received the BEACOP-DAC chemotherapy protocol (bleomycin, etoposide, cyclophosphamide, doxorubicin, vincristine, dacarbazine, and prednisone) for two cycles of 21 days. The reassessment finding shows a complete metabolic response. The patient completed the ABVD protocol (doxorubicin, bleomycin, vinblastine, dacarbazine) for four additional cycles of 28 days, with confirmation of complete remission at the end of treatment. The treatment was well-tolerated, with two episodes of uncomplicated febrile neutropenia during treatment with BEACOP-DAC.

The patient is seen regularly in consultation. She is still in complete remission one year after treatment.

## Discussion

Hodgkin's lymphoma (HL) was first described in 1932 by Thomas Hodgkin. It is a malignant hemopathy that represents 15 to 20% of lymphomas, characterized by the presence of Reed Sternberg cells, whose B lymphoid origin has been demonstrated. The cause of HL remains unknown [3]. There is an increased incidence in young adults as well as in patients aged 55 and older [4].

The disease is most often asymptomatic, discovered incidentally on a systematic X-ray, or revealed by mediastinal manifestations. About one-third of patients presented with systemic symptoms, including fever, night sweats, weight loss, and/or chronic pruritus [3,5]. The positive diagnosis is guided by the clinic and radiology, in particular, PET-FDG, and confirmed by histology that specifies the type of lymphoma [6].

Classic nodular sclerosis HL is the most common subtype, representing 50% to 80% of cases in most series [5]. It most frequently reaches adolescents and young adults involving the cervical, supraclavicular, and mediastinal regions [4]. The microscopic study shows cellular nodules surrounded by collagen bands arranged concentrically, which exhibit birefringence in polarized light [5].

In the case of the patient, the diagnosis was delayed by the etiological investigation of a possible infectious origin, a drug-related (corticosteroids) or inflammatory myopathy. Involvement of striated muscles in lymphomas is rare, it is observed in less than 1.5% of patients [7]. It may be of a primitive nature, or more often, linked to the invasion of the muscle from a neighboring adenopathy or a hematogenous dissemination of a pre-existing lymphoma [8]. In a literature review, Chim et al. identified 30 cases of primary muscular lymphoma [3]; these were mainly large B-cell lymphomas [9].

The main differential diagnosis of muscle lymphomas is acute sarcoidosis myositis, which results in a deterioration of general condition associated with proximal and symmetrical myalgias, affecting young patients with an average age of 37 years [10]. Myalgia can go as far as muscle contractures, with hypertrophy of the muscles concerned. Muscle weakness is rare [10].

MRI may be normal due to the small size of the lesions or may show muscle hyperintensity on diffuse T2 weighting [11]. MRI is more sensitive than CT for detecting muscle locations of lymphomas. It helps guide the muscle biopsy. In our case, the muscle biopsy was inconclusive. The diagnosis of certainty was based on the histological study of the biopsy of the mediastinal adenopathies, supplemented by PET-FDG in search of the hypermetabolism of the various sites in the whole body.

In addition, the MRI showed the bone involvement of the right femur. Bone lymphomas are rare, a distinction is made between primary bone lymphomas and secondary bone localizations of lymph node lymphoma. Belarbi et al. identified seven cases in a series, including six cases of primary bone lymphoma and one case of secondary bone lymphoma. The average age of primary bone lymphoma is 40 years [6].

In bone lymphomas, each bone can be a location for the development of lymphoma, but the femur is most commonly affected [2]. This distinguishes it from sarcoidosis involvement of the long bones, which is generally silent and predominant on the proximal and distal third, in particular on the forearm [12].

With regard to treatment, the histology and anatomical stage of the disease (limited or advanced) determine the initial choice of treatment. The presence of poor prognostic characteristics, constitutional symptoms, and the presence of a bulky mass define it as a single site of disease > 10 cm in diameter. Patients with early-stage disease are typically treated with combined modality strategies, using short cycles of combination chemotherapy followed by radiation therapy to the involved site, while those with advanced-stage disease receive longer chemotherapy, often without radiotherapy [4].

The prognosis for Hodgkin's lymphoma remains good, with an initial response rate exceeding 80% and high cure rates.

## Conclusions

This case highlights the importance of the combination of imagery and anatomopathological examination. This comprehensive approach not only facilitates a conclusive diagnosis but also helps in classifying the various types of lymphoma, which determines the most appropriate therapeutic management.
